# Risk factors for contracting watery diarrhoea in Kadoma City, Zimbabwe, 2011: a case control study

**DOI:** 10.1186/1471-2334-13-567

**Published:** 2013-12-02

**Authors:** Brian A Maponga, Daniel Chirundu, Notion T Gombe, Mufuta Tshimanga, Gerald Shambira, Lucia Takundwa

**Affiliations:** 1 Field Epidemiology Training Program, University of Zimbabwe, P.O. Box A178, Avondale, Harare, Zimbabwe; 2 Kadoma City Health Department, P.O. Box 460, Kadoma, Zimbabwe

**Keywords:** Watery Diarrhoea, Risk Factors, Kadoma City Zimbabwe

## Abstract

**Background:**

Kadoma City experienced an increase in watery diarrhoea from 27 cases during week beginning 5^th^ September, to 107 cases during week beginning 26^th^ September 2011. The weekly diarrhoea cases crossed the threshold action line during week beginning 5^th^ September at the children’s clinic in Rimuka Township, and the remaining four clinics reported cases crossing threshold action lines between week beginning 12^th^ September and week beginning 26^th^ September. Eighty-two percent of the cases were children less than 5 years old. We conducted a case controlstudy to determine risk factorsfor contracting watery diarrhoea in children less than 5 years in Kadoma City.

**Methods:**

An unmatched 1:1 case control study was conducted in Ngezi and Rimuka townships in Kadoma City, Zimbabwe. A case was a child less than 5 years old, who developed acute watery diarrhoea between 5^th^ September and 1^st^ October 2011. A control was a child less than 5 years old who stayed in the same township and did not suffer from diarrhoea. A structured questionnaire was administered to caregivers of cases and controls.Laboratory water quality tests and stool test results were reviewed.Epi Info™ statistical software was used to analyse data.

**Results:**

A total of 109 cases and 109 controls were enrolled. Independent protective factors were: having been exclusively breastfed for six months [AOR = 0.44; 95% CI (0.24-0.82)]; using municipal water [AOR = 0.38; 95% CI (0.18-0.80)]; using aqua tablets, [AOR = 0.49; 95% CI (0.26–0.94)] and; storing water in closed containers, [AOR = 0.24; 95% CI (0.07–0.0.83). The only independent risk factor for contracting watery diarrhoea was hand washing in a single bowl, [AOR = 2.89; 95% CI (1.33–6.28)]. *Salmonella, Shigella, Rotavirus,* and Enteropathogenic *Escherichia coli* were isolated in the stool specimens. None of the 33 municipal water samples tested showed contamination with *Escherichia coli,* whilst 23 of 44 (52%) shallow well water samples and 3 of 15(20%) borehole water samples tested were positive for *Escherichia coli.*

**Conclusions:**

The outbreak resulted from inadequate clean water and use of contaminated water. Evidence from this study was used to guide public health response to the outbreak.

## Background

Acute diarrhoea is defined as the passage of three or more loose or liquid stools per day, in a period not exceeding 14 days. Other types of diarrhoea include dysentery; and persistent diarrhoea which lasts 14 days or longer. Patients presenting with diarrhoea can also present with other symptoms such as vomiting, fever, and body weakness. Diarrhoea causes loss of body fluid and electrolytes, which can result in dehydration. If dehydration is not corrected, death can result [1].

The causes of diarrhoea can be bacterial, viral or parasitic. Bacteria causes of diarrhoea include *Vibrio cholerae, Escherichia coli, Campylobacter Jejuni, Salmonellae* and *Shigella* species. Viral causes of diarrhoea include rotavirus, adenovirus, and corona viruses. Parasitic causes of diarrhoea include *Giardia, Entamoeba, Cryptosporidium* and the helminthes (Strongyloides, Schistosoma) [2].

Diarrheal disease is a leading cause of child mortality and morbidity in the world, and mostly results from contaminated food and water sources.In developing countries, children below 3 years experience on average 3 episodes of diarrhoea every year. Globally, there are about 2 billion cases of diarrhoeal disease every year [1]. Fifteen percent of child deaths are directly attributable to diarrhoeal diseases [3]. According to the World Health Organization (WHO), key measures to prevent diarrhoea include access to safe drinking-water, improved sanitation, exclusive breastfeeding for the first six months of life, good personal and food hygiene and health education about how infections are spread [1].

### Background of the setting: Kadoma City

In 2011, Kadoma City had an estimated 100,000 inhabitants. Of these 13,200 were children less than 5 years old based on the 2002 census projections [4]. More than half the population resides in the city’s Rimuka Township. Nearly the whole population in Kadoma city has access to piped water. However, demand for water is very high, reaching as high as 60 mega litres per day, yet the city can only pump at best 25 mega litres per day [5]. The quantity of water pumped is further compromised by recurrent power cuts to the water treatment plants. In the absence of municipal water, residents use shallow wells and boreholes among others as water sources [5].

Kadoma City has had recurrent problems with watery diarrhoeal outbreaks since 2002. In 2007, a rotavirus outbreak killed 34 children [6]. A cholera outbreak in 2008 and 2009 affected 6,000 people, with 127 deaths [7]. Another cholera outbreak in 2010 affected 123 people and caused 4 deaths [8]. Most of the problems, such as shortage of water, and recurrent sewer blockages have not had permanent solution due to lack of financial resources.

**Figure 1:**
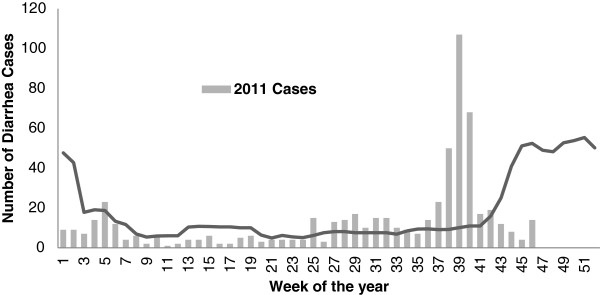
Weekly diarrhoeal threshold for family and child health clinic, Kadoma City, 2011.

In 2011, Kadoma City experienced an upsurge of watery diarrhoeal cases during week 36 (week beginning 5^th^ September), in 2011 (Figure 1). The total cases reported by the city’s five clinics doubled from 27 to 53 cases between week 35 and 36. The cases further doubled from 55 to 107 between week 37 and week 38. The weekly watery diarrhoeal cases at the under 5 clinic crossed the action threshold line during week 36 (Figure 1). The action threshold was defined using the C2 CUSUM method (defined as the sum of the mean plus three standard deviations for 7 preceding weekly diarrhea recordings, skipping two most recent weeks) [9]. The diarrhoeal cases at the Children’s clinic increased 7 fold, from 7 to 50 cases, between weeks 35 and 38.

The remaining four clinics (for all age groups) also experienced more than doubling of cases between week 35 and week 39. Initial analysis showed that about 85% of the cases were children less than five years old. The study was conducted to determine factors associated with contracting watery diarrhoea in children less than five years old.

## Methods

An unmatched 1:1 case–control study was conducted in Kadoma City’s Rimuka and Ngezi townships. The study was conducted among children less than 5 years old. A case was defined as a child less than five years old who presented to a health facility with acute watery diarrhoea, with or without vomiting and other symptoms, between 5^th^ September and 1^st^ October 2011, who had been resident in Kadoma City for one week prior to onset of symptoms. A control was defined asa child less than five years old, who did not develop diarrhoeabetween 5^th^ September 2011 and 1^st^ October 2011, and had been resident in Kadoma City, one week prior to the interview. Children whose caregivers agreed to participate were recruited into the study. Children who had passed diarrhoea for more than 14 days were excluded from participating in the study.

Epi Info™ statistical software was used to calculate sample size. Assuming 30% exposure in controls and 50% exposure in cases, using unchlorinated water, Odds Ratio of 2.4 [10], 95% confidence level, 80% power, and 10% refusal rate, we calculated a minimum sample size of 212 (106 cases and 106 controls).

Multistage sampling of cases was done. Rimuka and Ngezi townships were selected because they were the most affected. Using proportionate sampling, Rimuka was to provide 75(70%) cases whilst Ngezi was to provide 31(30%) cases. Cases were selected from clinic line lists; using random numbers generated using Microsoft™ Excel 2007. A control was selected from a household 10 houses away from a case. Where there was more than one child less than 5 years old at the household, a child whose age was closest to the age of the case was selected. If there was no child less than 5 years old, or if the caregiver declined to participate, the next house was selected till a less than 5 years old was obtained.

A pre-tested, interviewer administered questionnaire, was used to collect data from caregivers of cases and controls. The information collected included caregiver and case/control demographic information, symptoms, knowledge of caregiver on diarrhoea illness and treatment, duration of exclusive breast feeding, immunization status, the source of water, water treatment and storage, and sanitation facilities at the family home. The questions were in English and local Shona languages. Record review of water surveillance reports was done. The water quality parameters tested were bacteriological and chemical (chlorine content for municipal water, and pH). Bacteriological water quality tests had been done using a Delagua field testing machine. Water samples that were found with zero colonies after incubation for 24 hours were considered satisfactory. Residual chlorine content was tested using the Lovibond Comparator using DPD 1 tablets (N,N Diethyl-1.4 Phenylenediamine sulphate) to check colour changes. Specimens within the range 0.2 to 0.5 parts per million (ppm) were considered satisfactory. The pH was measured with the same equipment using phenol red tablets. Specimens with pH ranging between 6.5 and 8.5 were considered satisfactory. The results were reported as proportions.

Data were analyzed using Epi Info™. Means, proportions, frequencies, odds ratios and Chi square tests at 5% significant levels were generated using the software. Stratified analysis and forward step-wise logistic regression analysis was used to control for confounding and effect modification.

Permission to conduct the study was obtained from the local health authorities. Ethical review was done at the Health Studies Office, University of Zimbabwe. Written informed consent was obtained from all study participants caregivers. Children or relative with diarrhoea found during interviews were referred to the nearest health facility for free treatment.

## Results

### Descriptive epidemiology

A total of 1,091 cases of watery diarrhoea were attended to between 5^th^ September and 2^nd^ November 2011. Of these, 994 (91%) were residents of Kadoma City. The crude attack rate was 105 per 10,000 people. Five hundred and fifty two cases (51%) were females. Six hundred and ninety three (64%) cases were children less than 5 years old. The attack rate among children less than 5 years old was 548 per 10,000 people, whilst for those above 5 years old, the attack rate was 50 per 10,000 people. Five cases, all children less than five years old died of diarrhoea. The crude case fatality rate was 0.46%. One child died in hospital, and four children died at home after having sought treatment at health facilities.

All the residential areas in Kadoma City were affected. The highest risk of developing diarrhoea was in Ngezi Township, with an attack rate of 230 per 10,000 people, followed by Rimuka Township, with an attack rate of 102 per 10,000 people. However, during the early part of the outbreak, the highest risk of contracting diarrhoea was in Rimuka Township.

**Figure 2:**
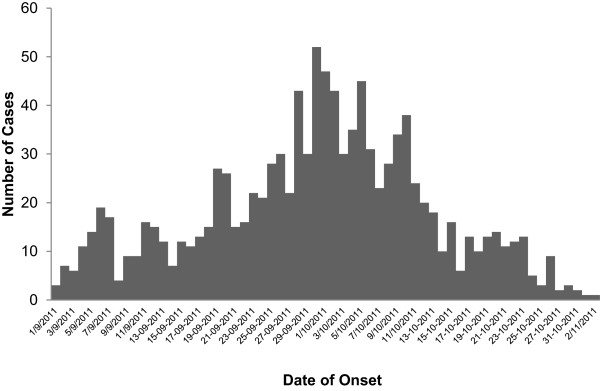
Epidemic Curve, Kadoma City, 1st September-3rd November, 2011.

Figure 2 shows the epidemic curve for Kadoma City for the period 1^st^ September to 3^rd^ November 2011. The outbreak commenced during the week beginning 5^th^ September. The epidemic curve shows multiple waves with progressively taller peaks that are 3–5 days apart up to the 1^st^ of October. The peaks became progressively shorter, until less than 5 cases were attended to per day from 23^rd^ October 2011.

### Stool specimen testing results

A total of 51 stool specimens were submitted to the local laboratory. Thirty specimens yielded *E.Coli,* 7 yielded *Shigella* Species, 3 yielded *Salmonella* species and one yielded Rotavirus. One out of the sixteen specimens submitted to the National Virology Reference Laboratory tested positive for Rotavirus. Two of the 10 specimens/isolates submitted to the National Microbiology Reference Laboratory (NMRL) were confirmed as Enteropathogenic *Escherichia coli*. No *Cholera* or *Cryptosporidium* was isolated.

### Water quality test results

All 33 municipal water samples tested were below the threshold limit for contamination with *Escherichia coli*. Three of the 15 (20%) borehole water samples and 23 of the 44 (52%) shallow well samples were above the threshold for contamination with *Escherichia coli*. Thirty-two (80%) of the 48 municipal water samples tested for chlorine had chlorine levels within normal range. All the 48 samples had pH levels within normal range. The samples were collected and analyzed between 1^st^ September and 22^nd^ October 2011.

### Analytic epidemiology

A total of 109 cases and 109 controls were recruited into the study. There were no significant differences in the demographic characteristics of cases and controls as shown in Table 1.

**Table 1:** Demographic characteristics of cases and controls, Kadoma City, 2011

**Variable**		**Cases**	**Controls**	**p value**
		**n = 109 (%)**	**n = 109 (%)**	
Child sex:	Female	49(45)	54(49)	0.59
	Male	60(55.0)	55(51)	
Township of residence:	Rimuka	31(28)	31(28)	>0.99
	Ngezi	78(71)	78(71)	
Caregiver mother:	Yes	97(89)	98(90)	>0.99
	No	12(11)	11(10)	
Caregiver sex:	Female	108(99)	107(98)	>0.99
	Male	1(1)	2(2)	
Religion of caregiver:	Apostolic	30(28)	29(27)	0.51
	Pentecostal	42(39)	44(40)	
	Orthodox	31(28)	25(23)	
	Traditional	6(6)	2(2)	
Caregiver level of education:	Primary and below	12(11)	16(15)	0.51
	Secondary and above	97(89)	93(85)	
Occupation of caregiver:	Housewife	94(86)	95(87)	0.55
	Informal	12(11)	13(12)	
	Maid	2(2)	0(0)	
Median age:	(months)	12(Q_1_ = 9: Q_3_ = 20)	17(Q_1_ = 11; Q_3_ = 35)	

### Knowledge of caregivers

Less than 50% of cases and controls caregivers had received health education on diarrhoea six months prior to the outbreak. The knowledge on prevention and home treatment of diarrhoea was fair, and did not differ significantly between cases and controls as shown in Table 2.

**Table 2:** Knowledge of caregivers on diarrhea among cases and controls, Kadoma City, 2011

**Knowledge attribute**	**Cases**	**Controls**	**Odds ratio**	**p- value**
	**n = 109 (%)**	**n = 109 (%)**	**(95% CI)**	
Received health education on diarrhoea in the last 6 months	46(43)	46(43)	0.98(0.57-1.69)	0.94
Correctly mentioned how to make salt and sugar solution	57(53)	70(64)	0.62(0.36-1.07)	0.12
Mentioned boiling water to prevent diarrhoea	70(64)	76(70)	0.78(0.44-1.37)	0.42
Mentioned using aqua tablets to prevent diarrhoea	72(66)	73(67)	0.96(0.55-1.68)	>0.99
Mentioned washing hands all the time after toilet	70(64)	74(68)	0.85(0.48-1.49)	0.67
Mentioned hand washing before handling food	64(59)	65(60)	0.96(0.56-1.65)	>0.99

### Risk factors for contracting diarrhoea

Statistically significant risk factors of contracting diarrhoea in children under 5 years were: family sourcing water outside home [OR = 2.7, 95% CI (1.11-6.79)]; hand washing in single bowl [OR = 2.95, 95% CI (1.47-5.89)]; garbage near home [OR = 1.98, 95% CI (1.04-3.77)] and; flies near home [OR = 1.76, 95% CI (1.02-3.02)] as shown in Table 3.

**Table 3:** Watery diarrhea risk factors among children less than five years old in Kadoma City, Zimbabwe, 2011

**Factor**		**Cases**	**Controls**	**Odds ratio**	**p value**
		**n = 109 (%)**	**n = 109 (%)**	**(95% CI)**	
Exclusive breastfeeding for 6 months	Yes	38(38)	58(56)	0.47(0.27-0.82)	0.01
	No	63(62)	45(44)		
Always use municipal water source	Yes	17(16)	37(34)	0.36(0.19-0.69)	0.03
	No	92(84	72(66)		
Fetch water outside family home	Yes	9(8)	21(20)	2.7(1.11-6.79)	0.03
	No	100(91)	86(80)		
Distance of water source from home	>100 m	52(55)	35(40)	1.84(1.02-3.32)	0.06
	<100 m	42(45)	52(60)		
Use of chlorine containing tablets	Yes	60(55)	75(69)	0.55(0.31-0.95)	0.04
	No	49(45)	34(31)		
Prepare water by boiling	Yes	20(18)	34(31)	0.47(0.35-0.89)	0.03
	No	89(82)	75(69)		
Store water in bucket with lid	Yes	94(86)	104(95)	0.30(0.11-0.86)	0.03
	No	15(14)	5(5)		
Hand washing facility at home	Yes	74(71)	90(84)	0.47(0.04-0.91)	0.03
	No	30(29)	17(16)		
Hand washing in a single bowl	Yes	33(30)	14(13	2.95(1.47-5.89)	0.002
	No	76(70)	95(87)		
Garbage near home	Yes	32(30)	19(18)	1.98(1.04-3.77)	0.05
	No	75(70)	88(82)		
Flies near home	Yes	61(57)	46(43)	1.76(1.02-3.02)	0.05
	No	46(43)	61(57)		

Statistically significant protective factors against contracting diarrhoea were: using municipal water source [OR = 0.36, (95% CI, 0.19- 0.69)]; treating water with chlorine containing water purification tablets [OR = 0.55 (95% CI, 0.31-0.95)]; treating water by boiling (OR = 0.47, (95% CI, 0.35-0.89)]; storingwater in closedcontainer [OR = 0.30 (95% CI, 0.11-0.86)]; family having hand washing facility [OR = 0.47(95% CI, 0.04-0.91)] and; child having been exclusively breastfed for 6 months [OR = 0.47, (95% CI, 0.27-0.82)] as shown in Table 3.

### Multivariate analysis

Hand washing in a single bowl [AOR = 2.89, 95% CI (1.33-6.28)] was an independent risk factor for contracting diarrhoea. Independent protective factors against contracting diarrhoea were: using municipal water [AOR = 0.38, 95% CI (0.18-0.80)]; reating waterchlorine containing tablets [AOR = 0.49, 95% CI (0.26-0.94)]; storing water in closed container [AOR = 0.24, 95% CI (0.07- 0.83)] and; child exclusively breastfed for six months (AOR = 0.44, 95% CI (0.24-0.82)] as shown in Table 4.

**Table 4:** 4 Independent factors associated with contracting diarrhoea in children less than five years old, Kadoma City, Zimbabwe, 2011

**Factor**	**Adjusted odds ratio (95% CI)**	**p value**
Exclusive breast feeding for 6 months	0.44 (0.24-0.82)	0.01
Use of municipal water always	0.38 (0.18-0.80)	0.01
Use of aqua tablets to treat water	0.49 (0.26-0.94)	0.03
Storing water in a bucket with lid	0.24 (0.07-0.83)	0.02
Hand washing is a single bowl	2.89 (1.33-6.28)	0.01

## Discussion

This study sought to establish risk factors for contracting watery diarrhoea among children less than five years old in Kadoma City. The epidemic curve is typical of a propagated outbreak, highly suggestive of person-to-person transmission. The multiple peaks, 3 to 5 days apart, suggest the incubation period of the causative organisms to be between 3 to 5 days. The causative organisms that were isolated, (*Rotavirus*, *Salmonella, Shigella* and *Escherichia coli)* fit into the average incubation period of 3 to 5 days which is typical of faecal contamination [11].

The importance of hand washing practices in preventing diarrheal illness is highlighted in this study. Hand washing in a single bowl was found to be a risk factor for contracting watery diarrhoea. It is biologically plausible for infection to be spread from one person into the water, then to the next person. Having a hand washing facility at home was found to be protective as the facility encourages run to waste method of hand washing, especially after using the toilet. These findings are supported by several studies. A systematic review of several studies, estimated that appropriate hand washing with soap, could reduce the risks of severe intestinal infections and of shigellosis by up to 48% and 59%, respectively [12].

The water could have been contaminated by blocked sewer. The problem of blocked sewer had earlier been highlighted by Mangizvo R.V et al., 2009 [13]. This could explain the contamination of shallow wells and boreholes in Kadoma City, most probably through seepage. In Karachi, Pakistan, Khan *et al.* demonstrated that 60% of sewer influent samples were positive for Rotavirus, which was also identified in one of the cases in the outbreak in Kadoma City [14].

Contamination of water can occur at any stage from the source, to the point of use. The biological plausibility of storing water in a closed container, boiling and use of aqua tablets was demonstrated in this study. The use of chlorine containing materials have been made use of in situations of acute water shortage, where people tend to use unsafe sources of water, as reported by Lantagne D S, *et al.*, in 2003 [15].

In Kadoma City, in this epidemic, the use of municipal water was protective, compared to water from other sources (AOR = 0.38, p = 0.01). Similar findings were obtained in Harare by Gonese G, *et al.*, in 2008. (Risk Factors associated with salmonella outbreak in Budiriro Suburb, Harare City, Zimbabwe, 2008-Unpublished). The laboratory tests on municipal water which was of satisfactory bacteriological and residual chlorine levels support the findings. At the same time, families who accessed water from municipal supply are more likely to access water in the family home. This study demonstrated that distance of the water source from home increased risk of developing diarrhoea. This results in families accessing less than the required amount of water per day, compromising personal hygiene practices such as hand washing.

Exclusive breast feeding for 6 months was protective against developing diarrhoea. Exclusively breastfed children were protected as they were unlikely to take any other foods or fluids likely to be contaminated. This protective effect was less for children on mixed breastfeeding and weaned from their mother’s milk.The protective effect of exclusive breastfeeding had been demonstrated using different study designs, in different settings. In Belarus, Kramer M.S. *et al.*, 2001, reported that exclusive breast feeding reduced risk of diarrhoea by 40% [16]. In Guinea Bissau, Mølbalk K, *et al.*, 1996, reported that partial breast feeding, RR = 1.23(1.08-1.40), and no breast feeding RR = 1.37(1.37-1.91) increased risk of diarrhoea [17]. In Zimbabwe, a nutritional survey in 2010 reported that 5.8% of children were exclusively breast fed [18]. Thus, efforts to increase the prevalence of exclusive breast feeding could reduce the impact of diarrhoea on child morbidity and mortality, in Zimbabwe.

Limitations of this study included the following: Controls might have contracted the disease but not developed clinical symptoms leading to case ascertainment bias. This could have under estimated the strength of some associations. The observed protective effect of the water treatment tablets is self reported, thus may lead to bias. Some of the caregivers were interviewed more than two weeks after treatment, which could have resulted in recall bias. The study was conducted in children less than five years old, thus the results may not be generalizable to residents older than five years. Some agents isolated in the local laboratory, *Salmonella* and *Shigella* could not be confirmed by the national microbiology reference laboratory.

## Conclusions

The outbreak was propagated and affected all the residential areas in Kadoma City. Mixed etiological agents were responsible for the outbreak. Knowledge on prevention and home treatment of diarrhoea was fair and did not differ between caregivers of cases and controls. The importance of providing adequate safe water, appropriate hand washing, exclusive breast feeding and good environmental hygiene were demonstrated to be protective against contractingdiarrhoea. The results of the study provided guidance for policy makers in responding to the outbreak, and formulating strategies to improve child health.

### Public health actions taken

Based on the evidence from this study water purification tablets, municipal water trucking to residential areas, disinfection and repair of boreholes, and sewer unblocking was conducted in the affected communities. Health education on importance of practicing good personal hygiene, boiling and treating water with water purification tablets before drinking and long term benefits of exclusive breast feeding was conducted, and is ongoing. All municipal clinics now have weekly updated diarrhoea reports. The national electricity distribution company installed dedicated electricity power lines to the municipal water pumping stations to maintain continuous water pumping.

## Competing interests

The authors declare that they have no competing interests.

## Authors’ contributions

BAM: conception, design, acquisition, analysis and interpretation of data and drafting the manuscript. DC: conception, design, acquisition, analysis and interpretation of data and drafting the manuscript. NTG: conception, design, data collection, analysis, interpretation and reviewing of several drafts of the manuscript for important intellectual content. GS and LT :conception, design, reviewing of several drafts of the manuscript for important intellectual content. MT had oversight of all stages of the research and critically reviewed the final draft for important intellectual content. All authors read and approved the final manuscript.

## Pre-publication history

The pre-publication history for this paper can be accessed here:

http://www.biomedcentral.com/1471-2334/13/567/prepub
